# The evaluation of antimicrobial stewardship for bacterial meningitis in neonates

**DOI:** 10.1002/pdi3.30

**Published:** 2023-10-05

**Authors:** Xinsi Chen, Kun Feng, Yu Zhang, Yongming Wang, Qianqian Zhao, Ya Hu, Kaizhen Liu, Hong Wei, Ziyu Hua

**Affiliations:** ^1^ Department of Neonatology Children's Hospital of Chongqing Medical University Chongqing China; ^2^ National Clinical Research Center for Child Health and Disorders Chongqing China; ^3^ Chongqing Key Laboratory of Pediatrics Chongqing China; ^4^ Ministry of Education Key Laboratory of Child Development and Disorders Chongqing China

**Keywords:** antimicrobial stewardship, CNS infections, meningitis, neonate

## Abstract

Bacterial meningitis (BM) is potentially life threatening in neonates, but the duration of antibiotic therapy is not well established. We aimed to compare the efficacy and safety among neonates suffering from BM of a relatively shortened duration of antibiotic treatment to the currently recommended course. We did a retrospective cohort study in neonates (gestational age [GA] or corrected GA ≥35 weeks) diagnosed with BM. Neonates in the study group were assigned to withdraw the antibiotics on condition that they were clinically stable after taking sufficient antibiotics with normal serological inflammatory biomarkers, whereas the cerebrospinal fluid (CSF) indicators remain abnormal. Neonates in the control group were treated until both serological and CSF indicators returned to normal as recommended. The incidence of recurrent infection after the discontinuation of antibiotics and adverse drug reactions (ADRs) during hospitalization was measured. A total of 233 neonates were enrolled, of whom 160 were assigned to a shortened antibiotic duration and 73 were treated according to the current guidelines. Twelve patients (7.5%) relapsed in the study group, whereas 4 (5.5%) relapsed in the control group (*χ* ^2^ = 0.320, *p* = 0.572). The incidences of ADRs were similar in both groups (*p* > 0.05). The study indicates that antibiotics might be safely discontinued in neonates (GA ≥35 weeks) diagnosed with BM who are clinically stable or improving after antibiotic treatment and feature normal serological inflammatory markers, no severe complications, and no evidence of systemic infection, even if CSF parameters are not completely normal.

## Graphical abstract

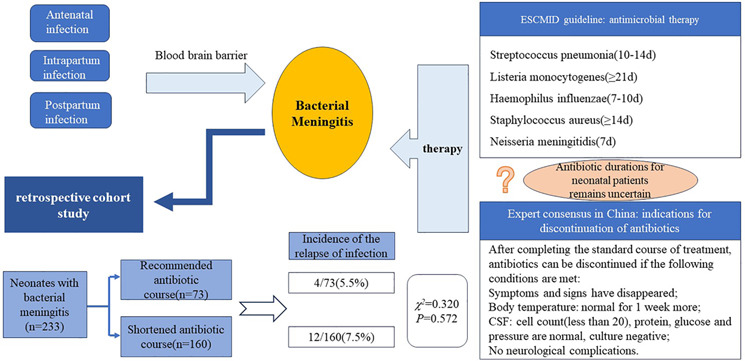

## 1 INTRODUCTION

Bacterial meningitis (BM) is a severe infectious disease in the neonatal period that features abnormal cerebrospinal fluid (CSF) parameters, and approximately a quarter of infected patients had elevated blood inflammatory indicators. Up to 24% of the BM patients who survive develop chronic neurological sequelae, such as hearing loss or focal neurological deficits.^1^ In addition, the mortality rates of neonatal BM range from 10% in high‐income countries to 58% in low‐income countries, with a disability incidence of 20%–50%.^2^, ^3^, ^4^ It was reported that febrile neonates have a higher rate of BM than infants in the second month of life.^5^ As the incidence of BM in young infants is associated with significant case fatality, the prevention strategies and guidelines to improve the early management of cases should be prioritized.^6^

The use of antibiotics is closely related to adverse drug reactions (ADRs) and accounts for up to 40.9% of ADRs, which are associated with increased morbidity and mortality as well as prolonged hospitalization. In a previous study, 20% (298/1488) of the patients experienced at least one antibiotic‐associated ADR.^7^, ^8^, ^9^ Unfortunately, it might cause more medications to be used among newborn patients.^10^, ^11^ However, antibiotics are critical therapies for infectious diseases that work well in the rapid sterilization of blood and CSF in most cases. Moreover, the duration of this treatment for BM is terribly long.

The distribution of pathogens differs with the patient's age and status of the immune system. As we know, group B *Streptococcus* (GBS), *Escherichia coli* (*E. coli*), and coagulase‐negative staphylococci are the leading pathogens of neonatal BM,^12^, ^13^ and the antibiotic duration that currently recommended differs with respect to the different types of pathogenic bacteria. For example, 14–21 days of antibiotics due to GBS or *Listeria*, 21 days due to gram‐negative bacteria or *Klebsiella pneumoniae*, and 21 days due to coagulase‐negative staphylococci.^14^ A recent update on community‐acquired BM based on the European Society of Clinical Microbiology and Infectious Diseases guideline and ongoing trial registries does not indicate any changes in the duration of antimicrobial therapy.^15^ A few studies show that shorter antibiotic treatment courses are also effective in treating BM in children (5 days vs. 10 days, 7 days vs. 10 days, 10 days vs. 14 days, etc.) without increasing the incidence of relapse of infection, neurological sequelae, or mortality.^16^, ^17^, ^18^, ^19^, ^20^ But the antibiotic duration for BM among neonates is not well established due to a lack of clinical reports, especially those with CSF culture‐negative meningitis, and the appropriate time to withdraw antibiotics remains controversial. In this study, a relatively shortened antibiotic duration was defined as the early discontinuation of the recommended antibiotic treatment according to the present consensus. To be more specific, the antibiotics were discontinued, even if the CSF parameters were still abnormal after receiving more than 14 days of treatment, on the condition that the neonates were clinically stable or improving, with normal serological inflammatory parameters (white blood cells count [WBCs], platelet count [PLT], immature/total neutrophil [I/T], C reactive protein [CRP], and procalcitonin [PCT]).

Whether an appropriate antibiotic regimen of a relatively shortened duration of neonatal BM treatment is as effective as the recommended duration has attracted increasing attention for years. The study aimed to assess and compare the effectiveness of different durations of antibiotic treatment in neonates' BM.

## 2 MATERIALS AND METHODS

### 2.1 Participants

A retrospective cohort study of shortened versus recommended antibiotic duration in neonates (gestational age [GA] or corrected GA ≥35 weeks) diagnosed with BM was performed by collecting and analyzing the medical records of patients admitted to the neonatal intensive care unit in our center, from January 1, 2015, to September 1, 2021. At admission, all the neonates with suspected BM received lumbar puncture (LP) for CSF examination of cells, glucose, protein, Gram stain, bacterial culture, and sensitivity; meanwhile, serological tests for inflammatory markers and cranial imaging examinations were performed.

### 2.2 Procedures

The hospitalized newborn infants who developed clinical signs of central nervous system (CNS) infection and fulfilled the requirement of a positive CSF culture or a positive CSF analysis were enrolled in the study. The positive CSF analysis in neonates was defined as the WBC ≥20 cells/mm^3^, microprotein >1.7 g/L, and glucose <2.2 mmol/L. And those who had combined congenital cardiovascular, gastrointestinal, and tracheal developmental diseases that might increase the use of antibiotics, lack of demographic data, already received more than 3 days of treatment before admission, and against‐advice discharge were excluded. According to the expert consensus on diagnosis and treatment of neonatal septicemia (*Chinese Journal of Pediatrics, 2019 edition*),^21^ a broad‐spectrum combination of antimicrobial agents is empirically used; the results of etiological and other nonspecific tests are pending. Penicillin plus the third generation cephalosporins were administered as soon as possible that targeted Gram‐positive bacteria and Gram‐negative bacteria. Antibiotics should be continued or switched to another depending on the results of bacteriological detection and the susceptibility test. Meningitis caused by GBS usually takes 14–21 days of antimicrobial therapy and 21 days or another 14 days after the CSF parameters returned to normal for meningitis caused by Gram‐negative bacteria. Neonates who had been treated with antibiotics (≥14 days) and whose CSF parameters (≥1 items) remain abnormal with no sign of systematic infection, were under complete assessment of the severity of illness and the possibility of withdrawal of antibiotics by clinicians. On that condition, the legal guardians were well informed of the risk of infection relapse if the antibiotics were discontinued prematurely and the risk of prolonged use of antibiotics (the ADR, prolonged hospitalization, nosocomial infection, etc.). Then, we received written consent from the guardians whether to choose to withdraw antibiotics or treat until full recovery such that the CSF parameters returned completely to normal as recommended. Subgroup analysis was performed between neonates with ≥2 serological abnormal markers (refer to the diagnostic criteria of neonatal sepsis^21^) and those with fewer than 2 abnormal markers. Once the antibiotics were discontinued in the study group, for the sake of security, a close observation of 2–3 days was done before discharge, and a CSF test was followed up 7–10 days later. During the outpatient follow‐up for neonates who were assigned to the fully recovered control group were also required to be hospitalized for CSF reexamination if they were wary of infection recurrence.

The primary outcome was the recurrence rate of infection, which was defined as the manifestations of infection with worsened serological inflammatory markers and CSF parameters, or a positive result of bacteriological examination. The secondary outcome was the incidence of ADRs, defined as the incidence of liver and kidney toxicity, rash, and fecal abnormalities. A follow‐up concerning the incidence of death, hearing loss, and neurological sequalae was recorded. Hearing was assessed with otoacoustic emission tests by trained nurses or with the auditory brainstem response if hearing loss was highly suspected. Unilateral or bilateral hearing impairment was defined as hearing loss. Intracranial color Doppler ultrasound was used for routine examination, and cranial magnetic resonance imaging scans were performed if there were suspected intracranial complications. The neonatal behavioral neurological assessment (NBNA) score, developmental quotient, amplitude‐integrated electroencephalogram, and video electroencephalogram (VEEG) were evaluated. Neonates who were not found or contacted were declared lost to follow‐up.

### 2.3 Statistical analysis

Statistical analysis was performed using *SPSS* version 22.0 software for Windows. Continuous variables were tested by using a Student's *t*‐test (mean [SD]) or the Mann–Whitney *U*‐test with a non‐Gaussian distribution (median [interquartile range, IQR]), and categorical variables were analyzed using Pearson's *chi*‐square test. Subgroup analysis was performed between groups that developed with <2 or ≥2 abnormal blood inflammatory parameters. A probability of less than 5% (*p* < 0.05) was considered significant.

## 3 RESULTS

**FIGURE 1:**
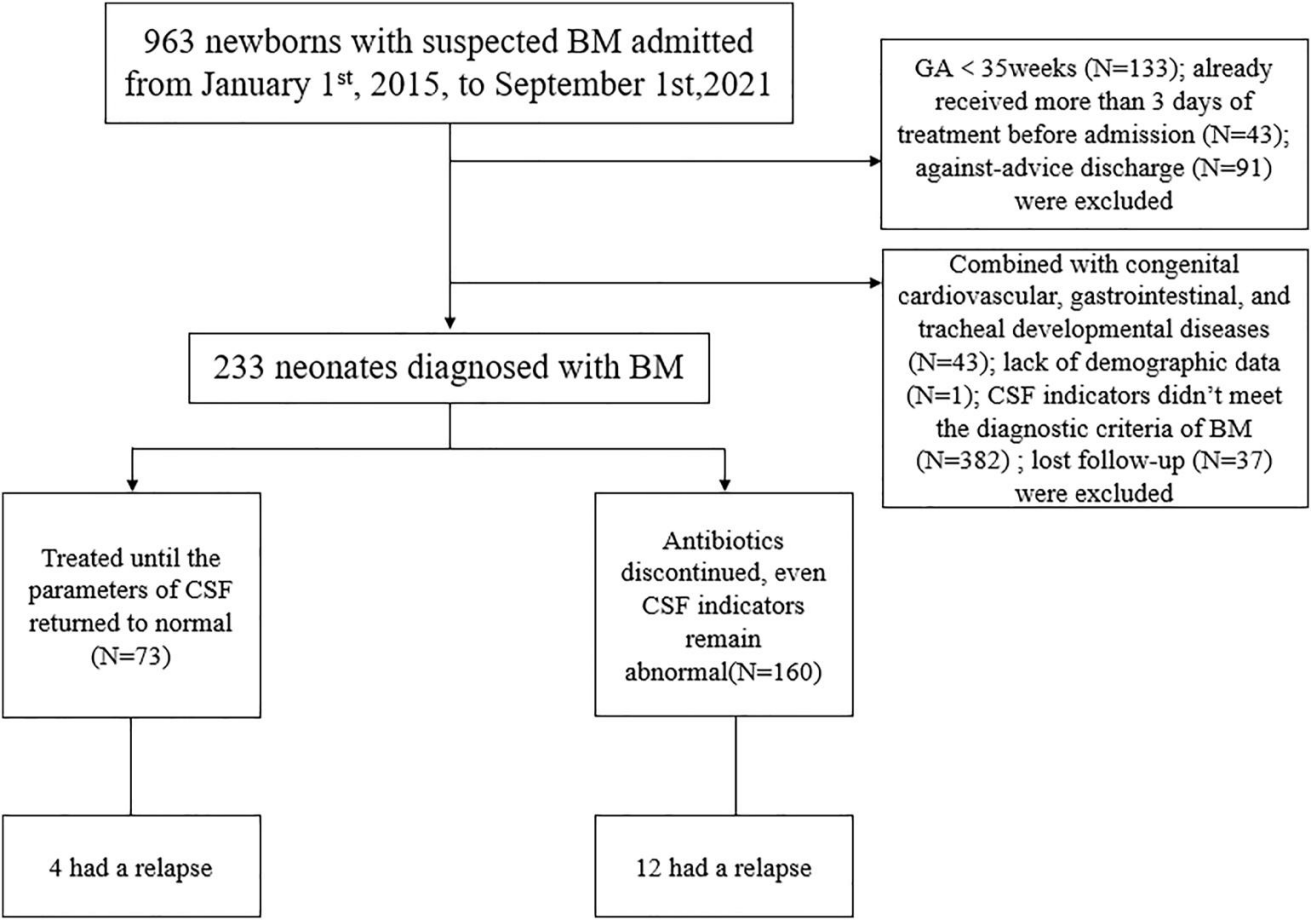
The flow diagram. The chart shows patient inclusion and exclusion in the study. BM, bacterial meningitis; CSF, cerebrospinal fluid; GA, gestational age.

A flowchart of participants in the study is presented in Figure 1. A total of 233 neonates diagnosed with BM were enrolled in the study. The study group and control group were comparable with reference to gender, GA, birth weight, the age of onset, premature rupture of membranes, cesarean delivery, amniotic fluid fecal stain, and intrapartum fever at admission (Table 1). Laboratory data of blood (I/T, PCT, and neutrophils percentage) were comparable in both groups at admission. The WBCs count was at a median of 14.44 × 10^9^/L (IQR 10.07–22.59 × 10^9^/L) in the control group and 12.17 × 10^9^/L (IQR 8.40–18.38 × 10^9^/L) in the study group (*p* = 0.036). The PLT was at a median of 283 × 10^9^/L (IQR 204.5–353 × 10^9^/L) in the control group and 323 × 10^9^/L (IQR 218.5–455.5 × 10^9^/L) in the study group (*p* = 0.023) (Table 2). In the control group, 4 neonates (5.5%) had recurrent infections after discharge, whereas 12 neonates (7.5%) in the study group had recurrent infections (*p* = 0.572). CSF parameters of the neonates in the study group were reassessed, of which 9 neonates (5.6%) were completely normal, 139 neonates (86.9%) improved but not to normal levels, and 12 (7.5%) worsened. Fourteen neonates (2 in the control group) developed subdural effusion. One of the neonates in the study group developed hydrocrania. No death occurred in this study group.

**TABLE 1:** Demographic data comparison.

	Control group (*n* = 73)	Study group (*n* = 160)	*χ* ^2^ */Z*	*p* value
Male [*n* (%)]	47(64.4)	94(58.7)	0.666	0.415
Gestational age [median (IQR), d]	274(268.5–279.5)	273(266–279.5)	−1.046	0.295
Birth weight [median (IQR), g]	3350(3062–3535)	3200(2907.5–3530)	−1.56	0.119
Age of onset [median (IQR), d]	8(1–15)	7(3–16)	−2.014	0.054
PROM [median (range), h]	0(0–48)	0(0–96)	−0.611	0.542
Cesarean delivery [*n* (%)]	35(47.9)	71(44.3)	0.258	0.612
Meconium‐staining amniotic fluid			0.722	0.679
0 [*n* (%)]	44(60.3)	87(54.4)		
1 [*n* (%)]	8(10.9)	21(13.1)		
2 [*n* (%)]	21(28.8)	52(32.5)		
Intrapartum fever			0.464	0.793
0 [*n* (%)]	59(80.8)	135(84.4)		
1 [*n* (%)]	13(17.8)	23(14.4)		
2 [*n* (%)]	1(1.4)	2(1.2)		

*Note*: “0” (no meconium‐staining amniotic fluid or intrapartum fever); “1” (having meconium‐staining amniotic fluid or intrapartum fever); “2” (unclear).

Abbreviations: IQR, interquartile range; PROM, premature rupture of membranes.

**TABLE 2:** Laboratory data comparison.

	Control group	Study group	*χ* ^2^ */Z/t*	*p* value
WBC [median (IQR), × 10^9^/L] [*n*]	14.4(10.1–22.6) [73]	12.2(8.4–18.4) [160]	−2.094	0.036
PLT [median (IQR), × 10^9^/L] [*n*]	283(204.5–353) [73]	323(218.5–455.5) [160]	−2.272	0.023
Neutrophils percentage [mean (SD)] [*n*]	0.64(0.15) [73]	0.61(0.14) [160]	1.471	0.143
I/T [median (IQR)] [*n*]	0.07(0.06–0.11) [56]	0.079(0.07–0.12) [110]	−0.803	0.422
Procalcitonin <0.5 ng/mL [*n* (%)]	33/97(34.0)	15/72(20.8)	0.020	0.888
CRP <10 mg/L [*n* (%)]	51/108(47.2)	34/77(44.1)	7.447	0.006
CSF examination^a^				
Nucleated cells [median (IQR), × 10^6^/L] [*n*]	4(1.5–8) [73]	5.5(2–14) [160]	−2.681	0.007
Microproteins [median (IQR), g/L] [*n*]	0.90(0.68–1.20) [73]	0.90(0.66–1.25) [160]	−0.716	0.474
Glucose [median (IQR), mmol/L] [*n*]	2.33(2.25–2.46) [73]	1.94(1.77–2.06) [160]	−11.54	<0.001

Abbreviations: CRP, C‐reactive protein; CSF, cerebrospinal fluid; IQR, interquartile range; I/T, immature neutrophil count/total neutrophil count; PLT, blood platelet; WBC, white blood cell.

^a^ The CSF parameters above were the last test before the withdrawl of antibiotics.

Before the antibiotics were discontinued, the CSF parameters were recorded and compared. Nucleated cells were lower in the control group than those in the study group, and the concentration of glucose in the control group was higher than that in the study group. The difference was statistically significant. Microproteins in the control group were at a median of 0.90 g/L (IQR 0.68–1.20 g/L) as compared to the study group, with a median of 0.90 g/L (IQR 0.66–1.25 g/L) (*p* = 0.474). Subgroup analysis was performed to compare the laboratory data and clinical outcomes. The concentration of glucose in the CSF was significantly different between the control and the study groups in subgroup analysis, and in the subgroup (≥2 abnormal serological inflammatory parameters), the nucleated cells in the CSF were at a median of 4 × 10^6^/L (IQR 2–8 × 10^6^/L) in the control group and 7 × 10^6^/L (IQR 2–17 × 10^6^/L) in the study group (*p* = 0.011) (Table 3).

**TABLE 3:** Subgroup analysis of CSF data.

	<2 abnormal serological inflammatory parameters				≥2 abnormal serological inflammatory parameters			
	Control group (*n* = 26)	Study group (*n* = 37)	*χ* ^2^/*Z*/*t*	*p* value	Control group (*n* = 47)	Study group (*n* = 123)	*χ* ^2^/*Z*/*t*	*p* value
Nucleated cells [median (IQR), × 10^6^/L]	3.5(1–10)	4(2–10)	−0.786	0.432	4(2–8)	7(2–17)	−2.538	0.011
Microproteins [median (IQR), g/L]	0.92(0.66–1.28)	0.79(0.61–1.13)	−1.075	0.282	0.90(0.68–1.09)	0.94(0.72–1.29)	−1.335	0.182
Glucose [median (IQR), mmol/L]	2.35(2.26–2.46)	2.00(1.82–2.10)	−6.159	<0.001	2.33 (2.25–2.47)	1.92 (1.77–2.04)	−9.588	<0.001

Abbreviations: CSF, cerebrospinal fluid; IQR, interquartile range.

In this study, *E. coli* was the main pathogen detected in CSF, accounting for 55% of the total positive cases, and the others that were detected were *Staphylococcus* (3/20), *Streptococcus pneumoniae* (1/20), *Streptococcus agalactiae* (1/20), *Pseudomonas stutzeri* (1/20), *Chryseobacterium meningosepticum* (2/20), and *Enterococcus faecium* (1/20). Among the 16 neonates with recurrent infection, 1 was infected by *S. pneumoniae* that identified in the CSF at first admission, 1 was infected by *P. stutzeri*, and the remaining 14 showed negative results (Table 4).

**TABLE 4:** Organisms identified at the time of enrollment by treatment.

	Control group (*n* = 73)	Study group (*n* = 160)	Total
Positive culture of cerebrospinal fluid	1(1.4%)	19(11.9%)	20(8.6%)
*E. coli*	0	11(6.9%)	11(4.7%)
*Staphylococcus*	1(1.4%)	2(1.2%)	3(1.3%)
*Streptococcus pneumoniae*	0	1(0.6%)	1(0.4%)
*Pseudomonas stutzeri*	0	1(0.6%)	1(0.4%)
*Chryseobacterium meningosepticum*	0	2(1.2%)	2(0.8%)
*Enterococcus faecium*	0	1(0.6%)	1(0.4%)
*Streptococcus agalactiae*	0	1(0.6%)	1(0.4%)
Gram stain of cerebrospinal fluid			
Gram‐positive cocci	0	1(0.6%)	1(0.4%)
Blood culture			
Positive for any study organism	12(16.4%)	52(32.5%)	64(27.5%)
Negative for any study organism	61(83.6%)	108(67.5%)	169(72.5%)
*E. coli*	5(6.8%)	23(14.4%)	28(12.0%)
*Staphylococcus*	3(4.1%)	10(6.2%)	13(5.6%)
*Streptococcus pneumoniae*	0	0	0
*Klebsiella pneumoniae*	2(2.7%)	1(0.6%)	3(1.3%)
*Streptococcus agalactiae*	1(1.4%)	6(3.7%)	7(3.0%)
*Enterobacter cloacae*	1(1.4%)	0	1(0.4%)
*Chryseobacterium meningosepticum*	0	1(0.6%)	1(0.4%)
*Haemophilus influenzae type B*	0	7(4.4%)	7(3.0%)
*Citrobacter*	0	1(0.6%)	1(0.4%)
*Edwardsiella tarda*	0	1(0.6%)	1(0.4%)
*Enterococcus faecium*	0	2(1.2%)	2(0.8%)

The comparison of the recurrence of infection had no statistical significance in the two subgroups (*p* = 0.086 and 0.243, respectively), and the same result was found for the comparison of the occurrence of ADR (abnormal liver function, abnormal renal function, fecal abnormalities, and rash) between the groups (Table 5). For the subgroup within 2 abnormal inflammatory markers in the blood, antibiotics were administered for a median of 16.5 days (IQR 14–20 days) in the control group and 17 days (IQR 15–21 days) in the study group (*p* = 0.421). For the subgroup ≥2 abnormal inflammatory markers in the blood, antibiotics were administered for a median of 17 days (IQR 15–21 days) in the control group and 23 days (IQR 16–31 days) in the study group (*p* = 0.002). Neonates in the two subgroups that experienced ADR had no statistical difference (Table 6).

**TABLE 5:** Clinical outcomes comparison.

	Control group	Study group	*χ* ^2^ */Z*	*p* value
Antibiotic duration [median (IQR), d] [*n*]	17(14.5–21) [73]	21(16–28) [160]	−3.413	0.001
Hospital stay [median (IQR), d] [*n*]	18(15–22.5) [73]	23(16–29.8) [160]	−3.276	0.001
Recurrent infection [*n* (%)]	4/73(5.5)	12/160(7.5)	0.320	0.572
Abnormal liver function [*n* (%)]	7/73(9.6)	9/160(5.6)	1.232	0.267
Abnormal renal function [*n* (%)]	4/73(5.5)	12/160(7.5)	0.320	0.572
Fecal abnormalities [*n* (%)]	17/73(23.3)	65/160(40.6)	6.607	0.010
Rash [*n* (%)]	12/73(16.4)	33/160(20.6)	0.564	0.453

Abbreviation: IQR, interquartile range.

**TABLE 6:** Subgroup analysis of clinical outcomes.

	<2 abnormal serological inflammatory parameters				≥2 abnormal serological inflammatory parameters			
	Control group (*n* = 26)	Study group (*n* = 37)	*χ* ^2^/*Z*/*t*	*p* value	Control group (*n* = 47)	Study group (*n* = 123)	*χ* ^2^/*Z*/*t*	*p* value
Antibiotic duration [median (IQR), d]	16.5(14–20)	17(15–21)	−0.805	0.421	17(15–21)	23(16–31)	−3.059	0.002
Hospital stay [median (IQR), d]	17(14.8–21.5)	17(15.0–23.5)	−0.316	0.752	19(15–23)	24(17–33)	−3.145	0.002
Recurrent infection [*n* (%)]	2/26(7.7)	0/37(0)	2.939	0.086	2/47(4.2)	12/123(9.7)	1.362	0.243
Abnormal liver function [*n* (%)]	2/26(7.7)	0/37(0)	2.939	0.086	5/47(10.6)	9/123(7.3)	0.496	0.481
Abnormal renal function [*n* (%)]	2/26(7.7)	3/37(8.1)	0.004	0.952	2/47(4.2)	9/123(7.3)	0.527	0.468
Fecal abnormalities [*n* (%)]	4/26(15.4)	12/37(32.4)	2.342	0.126	13/47(27.6)	53/123(43.1)	3.409	0.065
Rash [*n* (%)]	3/26(11.5)	7/37(18.9)	0.623	0.430	9/47(19.1)	26/123(21.1)	0.082	0.774

Abbreviation: IQR, interquartile range.

The NBNA (<35 scores), test of infant motor performance (mild to severe), aEEG, VEEG, brain‐stem auditory evoked potential (detected as abnormal), and hearing test (hearing loss) results were compared between the two groups, and the differences were not found to be statistically significant (*p* = 0.976, 0.948, 0.908, 0.540, 0.577, and 0.134, respectively) (Table 7).

**TABLE 7:** Neurodevelopmental assessment comparison.

	Control group	Study group	*χ* ^2^	*p* value
Hearing deficit [*n* (%)]	7/68(10.3)	25/136(18.4)	2.242	0.134
aEEG [*n* (%)]	12/36(33.3)	32/93(34.4)	0.013	0.908
VEEG [*n* (%)]	6/13(46.1)	12/33(36.4)	0.375	0.540
BAEP [*n* (%)]	0/3(0)	2/21(9.5)	0.312	0.577
NBNA [*n* (%)]	13/47(27.6)	24/86(27.9)	0.001	0.976
TIMP [*n* (%)]			0.361	0.948
0	1/16(6.2)	5/51(9.8)		
1	4/16(25.0)	10/51(19.6)		
2	9/16(56.2)	30/51(58.8)		
3	2/16(12.5)	6/51(11.8)		

*Note*: “0” (normal); “1” (*M* − 1SD ∼ *M* − 0.5SD, mildly abnormal); “2” (*M* − 2SD ∼ *M* − 1SD, moderately abnormal); “3” (<*M* − 2SD, severely abnormal).

Abbreviations: aEEG, amplitude integrated electroencephalogram; BAEP, brain‐stem auditory evoked potential; NBNA, neonatal behavioral neurological assessment; TIMP, test of infant motor performance; VEEG, video electroencephalogram.

## 4 DISCUSSION

The findings show that the efficacy of shortened antibiotic treatment was not inferior to the recommended course in the neonates with BM in whom the clinical condition was stable and improving. Early diagnosis and prompt use of antibiotics are probably more important in reducing the mortality and morbidity than an extended antibiotic treatment regimen. Antibiotics are critical for anti‐infective therapy, while the duration of antibiotic use is terribly long for severe infectious diseases, resulting in the occurrence of more ADR. For neonates, off‐label drug use is widespread because only 9% of the approved drugs are licensed for newborns, and antibiotics are the most frequent off‐label drugs used. Therefore, limiting the usage of antibiotics might assist in reducing the occurrence of ADR.^10^ Besides, bacterial drug resistance has become a serious public health problem that attracts extensive attention worldwide. Overuse and misuse of antibiotics could lead to antimicrobial resistance. According to the WHO report of antibiotic consumption, at least two million patients in the United States were infected with antibiotic‐resistant bacteria each year, and more than 23,000 people died from infection. Neonates are vulnerable to severe infectious diseases including BM and have much higher morbidity than other age groups.^22^ A few studies showed that a shortened duration of antibiotics had no less efficacy than the recommended duration of therapy in children (≥1 month)^16^, ^17^, ^18^, ^19^; unfortunately, the antibiotic therapy is not well established in neonates for the lack of research. Clinicians usually struggled to decide when to withdraw antibiotics if the neonatal patients were clinically stable with sufficient antibiotic duration and with normal serological inflammatory parameters, whereas the CSF parameters were still abnormal. Prematurely discontinuation of antibiotics might increase the risk of infection recurrence, while prolonged use of it might lead to more ADR and drug resistance. The appropriate antimicrobial duration to treat neonatal BM, especially bacterial culture‐negative patients remain uncertain.

Bacterial culture of CSF is diagnostic for BM; however, the positive rate is poorly low (20 neonates (8.6%) in the study) as 10.7% of the neonates had already received antibiotic treatment prior to being transported to our center. Statistically, the positive rate of CSF culture (19/160 (11.9%) in the study group and 1/73 (13.7%) in the control group) was lower than that of blood culture (52/160 (32.5%) in the study group and 12/73 (16.4%) in the control group). Consequently, for culture‐negative meningitis, CSF parameters, including the cell count and the concentrations of protein and glucose are powerful predictors of CNS infectious diseases.^23^ The high concentration of protein might predict a poor outcome in newborns with BM.^24^ Classic abnormalities of CSF composition in BM are the pleocytosis of mainly polymorphic leukocytes, low glucose concentration, a low CSF‐to‐blood glucose ratio, and elevated concentration of protein levels.^25^ Necessarily, these parameters were reexamined to evaluate the therapeutic efficacy. Based on moderate evidence, pretreatment does not adversely affect the CSF cell count, but pretreatment does affect the positive test result, especially for meningococcal meningitis. It is necessary to distinguish and carefully evaluate whether traumatic LP has been performed, which results in increased CSF protein levels and leukocytes.^26^, ^27^ In this study, the concentration of glucose tested before the discontinuation of antibiotics were obviously lower in the study group than that in the control group, whereas the recurrence rate of infection, the incidence of ADR, and the neurological function between the two groups were not significantly different.

For the patients who had a good response to antibiotics and whose clinical condition was stable or improved gradually, ampicillin combined with third‐generation cephalosporins was the empirical treatment. Cephalosporins are widely used for their advantage in reducing drug resistance and their efficacy in sterilization. For severe cases of BM, vancomycin, often in combination with meropenem, is recommended. In addition, specific targeted treatment was performed once the pathogen was identified.^13^, ^28^ Safety and efficacy should be considered before withdrawing antibiotics.

In total, 16 neonates had recurrent infection; the incidence of recurrent infection in the study group and control group were 7.5% and 5.5%, respectively (*p* = 0.572). They had worse CSF parameters than those detected before discontinuing antibiotics. Among the 16 neonates who suffered from recurrent infection, 13 patients manifested with fever, 3 with convulsion, and 2 with both fever and intracranial hypertension (manifested as increased tension of anterior fontanelle). The neurological function of the neonates was assessed as well. Hearing deficit is one of the most frequently reported sequelae of BM and often occurs in patients with pneumococcal meningitis.^29^ The incidence of hearing deficit in both groups was similar in this study (*p* = 0.134).

Neonatal BM ranges in severity. For the 233 enrolled neonates, serological parameters (WBC, I/T, CRP, PCT, and PLT) differed, and the more abnormal the parameters were, the more severe the infection was as any two or more serological inflammatory parameters that meet the criteria (WBC ≥20 × 10^9^/L [age ≥3 days] or WBC ≥30 × 10^9^/L [6 h < age <3 days] or WBC <5 × 10^9^/L [at any age]; CRP ≥10 mg/L; PCT ≥0.5 ng/mL [age >3 days]; PLT <100 × 10^9^/L; I/T ≥ 0.16 [within 3 days] or I/T ≥ 0.12 [age >3 days]) could be clinically diagnosed with neonatal sepsis.^21^ In the study, neonates with ≥2 abnormal parameters in blood were assigned to the more severe group, allowing subgroup analysis. The study showed that the time of antibiotic use in the study group was not shorter than that in the control group for neonates because the individuals might not be uniformly sensitive to antibiotic treatment, and that was why the CSF parameters remained abnormal for a longer time. But on its own, the course of antibiotics was still shorter than recommended therapy. The length of hospital stay was closely related to antimicrobial therapy or even longer than antibiotic duration as the clinicians kept close watch on the study groups for at least 24–48 h after withdrawing antibiotics, in order to observe whether there were any signs of recurrent infection.

For neonatal BM, the lack of clinical research about antibiotic duration made it difficult to create clear guidelines. It is unethical to randomly assign neonates regardless of the severity of disease as intracranial infection might worsen or increase the risk of complications, neurological sequelae, and death if antibiotics are discontinued in an inappropriate way. Thus, the findings are limited in regard to guiding therapy for all cases of neonatal BM. However, this study did consider the safety and efficacy of the treatment strategy, and a subgroup analysis in the assessment of efficacy could take full advantage of data and eliminate confounding factors. Even though the overall outcome in the study was not harmful for the neonates, the sample size was insufficient for the assessment of separate pathogens as the organisms were not specifically restricted in the study. As a tertiary referral center, the intact clinical information of admitted neonates could not be obtained because some of the patients had been admitted to local hospitals and had already received antibiotics prior to transfer to our department. These are common problems usually encountered in retrospective cohort studies. Future studies may include more cases and analyze the outcomes with different pathogens detected in the CSF. If possible, a long‐term follow‐up of growth and intelligence of the neonates could be recorded.

In conclusion, the shortened duration of antibiotics for uncomplicated cases of neonatal BM might be safe, as a relatively shorter course of antibiotic treatment was no less effective than that of the present recommended regimen. Hopefully, this study will assist in reducing off‐label drug use and possibly minimize drug resistance. Further multicenter trials should be conducted to validate the results.

## AUTHOR CONTRIBUTIONS

All authors contributed to the study conception and design. Material preparation, data collection, and analysis were performed by Xinsi Chen, Kun Feng, and Ziyu Hua. The first draft of the manuscript was written by Xinsi Chen, and all authors commented on previous versions of the manuscript. All authors read and approved the final manuscript.

## CONFLICT OF INTEREST STATEMENT

The authors have no relevant financial or nonfinancial interests to disclose.

## ETHICS STATEMENT

This study received ethics approval from the Institutional Review Board of the Children's Hospital of Chongqing Medical University (approval No. 2019‐268‐2).

## CONSENT FOR PUBLICATION

Not applicable.

## Data Availability

The data that support the findings of this study are available from the corresponding author upon reasonable request.
